# Patient-Reported Symptom Changes Following Structured Intake Advice for Multivitamin-Associated Nausea After Bariatric Surgery

**DOI:** 10.7759/cureus.108473

**Published:** 2026-05-08

**Authors:** Ingrid Kruizinga, Aude Pereira, Hendrika Smelt

**Affiliations:** 1 Medical Affairs, FitForMe B.V., Rotterdam, NLD; 2 Customer Service, FitForMe B.V., Paris, FRA; 3 Surgery, Elkerliek Hospital, Helmond, NLD

**Keywords:** adherence, bariatric surgery, compliance, intake instructions, multivitamin supplementation, nausea, tolerability

## Abstract

Introduction

Adherence to post-bariatric supplement intake may be affected by tolerability issues. This study aimed to characterize nausea associated with supplement intake and explore the patient-reported symptom course following structured intake instructions in a real-world customer-support context.

Methods

This retrospective observational study with prospectively collected data consisted of two parts. Part one: a cross-sectional survey of 540 FitForMe customers after bariatric surgery who reported nausea, assessing the timing of supplement intake, the onset and triggers of nausea, and the impact on daily functioning. Part two: a single-arm, uncontrolled follow-up of a separate sample of 75 French FitForMe customers experiencing nausea, who received structured intake instructions during standardized telephone interviews. Recommendations included switching from capsules to chewable tablets, taking supplements with meals, dividing doses, and allowing slower dissolution. The presence of nausea (yes/no) was reassessed by telephone one week later.

Results

Part one indicated that a large percentage of individuals did not take the supplement according to intake instructions; 256 (47.4%) took it on an empty stomach. Nausea occurred in 93 patients (17.2%) before the actual intake of the supplement. Overall, 365 patients (67.6%) reported a score of 5 or higher on a 0-10 scale measuring the impact on daily life, where 0 indicated no restriction, and 10 indicated being unable to do anything.

In part two, at the one-week follow-up, 55 of 75 patients (73.3%) reported resolution of nausea (within-patient comparison, p<0.001). The recommendation most frequently endorsed by patients as perceived to have contributed to their symptom course was switching from capsule to chewable tablet, reported as helpful by 41 of 44 patients who made this switch (93.2%; 54.7% of the total follow-up cohort, n=75; p<0.001). Taking supplements with lunch or dinner was also commonly endorsed (n=42, 56.0%). Additional complaints were common, including potentially dumping-related symptoms (n=30, 40.0%), food intolerance (n=28, 37.3%), and altered taste or smell (n=31, 41.3%).

Conclusion

In a selected sample of bariatric patients who reported nausea during multivitamin use, incorrect intake practices and multifactorial symptom patterns were common. In a follow-up cohort, structured intake advice was associated with patient-reported resolution of nausea in many participants. While these observational findings do not establish causality, the recommendations are simple, low-cost, and easy to implement, and may be considered as a pragmatic first step in routine post-bariatric care.

## Introduction

Metabolic bariatric surgery (MBS) is widely recognized as one of the most effective long-term interventions for achieving significant and sustained weight loss in individuals with obesity. However, despite its clinical success, MBS also presents distinct nutritional challenges [1-3].

One of the most prevalent complications following MBS is the development of micronutrient deficiencies. To mitigate this risk, clinical guidelines strongly recommend lifelong daily use of a specialized multivitamin tailored to the specific needs of bariatric patients [1-3]. Such supplementation is critical to maintaining adequate micronutrient status and preventing clinical complications, such as anemia, neurological symptoms, bone demineralization, and fatigue.

However, adherence to daily multivitamin use after MBS is often suboptimal, with estimates ranging from 20% to 32% [4-7], depending on the population, follow-up duration, and type of procedure.

Several factors contribute to poor supplement adherence in this population. These include forgetfulness, cost, pill burden, lack of perceived need, and gastrointestinal side effects [5,6]. Smelt and colleagues found that gastrointestinal complaints that are directly related to multivitamin supplementation (MVS) intake were reported by 58.5% of the people who no longer used supplements. The most frequently reported complaint was nausea (85.4%) [5].

To address this issue, a better understanding of nausea-related complaints in the context of supplement use after MBS is needed. While nausea is commonly reported, detailed insights into its onset, triggers, and impact on adherence are still limited. Furthermore, simple, practical interventions, such as optimizing intake instructions, may offer a low-threshold opportunity to reduce side effects and thereby support adherence.

The objective of this study was twofold. First, to explore potential contributing factors and characteristics of nausea associated with MVS after MBS, including its onset, triggers, and impact on daily functioning, in a selected sample of patients who contacted FitForMe customer service due to nausea. Second, to explore patient-reported symptom changes following structured intake instructions, including the perceived helpfulness of recommendations, in a cohort of patients from a different selected sample who also contacted FitForMe customer service due to nausea.

By identifying modifiable factors that influence tolerance, this study aims to support the development of practical strategies to improve supplement adherence and, ultimately, prevent micronutrient deficiencies in the long term.

## Materials and methods

Study design

All data were collected by FitForMe B.V., a company that develops and offers specialized supplementation for people with obesity or those undergoing weight loss management, including MBS. Data analysis and interpretation were performed in the Netherlands. The independent statistical analysis was conducted by H.J.M. Smelt at Elkerliek Hospital (Helmond, The Netherlands), and the manuscript was drafted jointly with the authors based at FitForMe B.V. (Rotterdam, The Netherlands).

This retrospective observational study with prospectively collected data consisted of two parts. The first was a cross-sectional survey among customers who reported nausea related to MVS. The second part involved routine customer-service provision of structured intake instructions with short-term follow-up of patient-reported symptoms.

Part one: survey

Study Population and Data Collection

An online survey was sent out to 2,534 customers who had been in contact with FitForMe regarding nausea complaints associated with supplement intake. Eligible participants were FitForMe customers who had undergone sleeve gastrectomy or Roux-en-Y gastric bypass, were currently using a FitForMe multivitamin supplement (i.e., WLS Optimum or WLS Forte), and had contacted the FitForMe customer service because of nausea. A total of 540 customers (21.3%) completed the questionnaire. Data collection took place between November 2018 and September 2019. The questionnaire was distributed via email to FitForMe customers from eight commercial regions covering 10 European countries (The Netherlands, Belgium, France, the DACH region (Germany, Austria, and Switzerland), Italy, Spain, Portugal, and Poland) who had contacted the FitForMe customer service because of nausea.

Materials

The survey (Appendix A) was developed by the authors to assess the onset, characteristics, and impact of nausea potentially related to supplement intake. It consisted of structured questions divided into two categories: (1) timing, which addressed when the supplement use and nausea began and the daily intake routine, (2) nausea, covering the perceived cause, symptom description, and impact on daily life. In addition, demographic and product-related variables (country/region, type of bariatric surgery (sleeve gastrectomy or Roux-en-Y gastric bypass), and supplement type (capsule or chewable tablet) were retrieved from the FitForMe customer database and linked to the survey responses through the customer identifier. Linkage was performed before anonymization; the dataset analyzed for the present publication was fully anonymized.

The complete survey is presented in Appendix A. The present analysis reports the items on the timing of supplement initiation, timing of supplement intake during the day, onset of nausea complaints, nausea triggers, timing of nausea around intake, restriction of daily life on a 0-10 scale, and overall impact description. The free-text item describing the nausea sensation, items on coping strategies, and what worked best for the individual respondent, and the open-ended fields on patient suggestions and additional comments were collected for service-evaluation purposes within FitForMe's customer support and were not analyzed for the present publication.

Analyses

Descriptive statistics were used to calculate all counts and percentages using IBM SPSS Statistics version 29.0.2.0 (released 2022; IBM Corp., Armonk, New York, United States).

Part two: follow-up after intake advice

Population and Data Collection

These data were collected in France by the French customer service of FitForMe in a different sample from the patients in part one. Eligible participants were FitForMe customers who had undergone a sleeve gastrectomy, who were currently using a FitForMe multivitamin supplement (e.g., WLS Optimum), and had proactively contacted customer service to report nausea that they attributed to supplement use. Between August and November 2023, contact was established with 116 customers. Participants were interviewed about their experience with nausea. Subsequently, in the same conversation, they received standard intake instructions, and if they were using the capsule formulation, they received a trial pack of chewable tablets. One week later, participants were contacted again by phone to evaluate whether their nausea symptoms had resolved. Patients who could not be reached at the scheduled follow-up time, despite repeated attempts, were considered lost to follow-up and excluded from the follow-up analysis. No additional reasons for non-response (e.g., refusal, discontinuation of supplement use) could be recorded.

All calls were conducted by experienced registered dietitians employed (>five years) by FitForMe. Patient responses were recorded directly in MS Excel (Microsoft; Redmond, WA, USA) using predefined response categories.

Materials

A structured telephone script (Appendix B) was developed by the authors for service evaluation purposes and included both closed and open-ended questions. It was designed for use at two time points:

First contact: to assess the possible associated factors of nausea related to multivitamin use after MBS. Topics included the onset of nausea, an open-ended item asking participants to describe their nausea symptoms in their own words, and potential contributing factors, such as food intolerance, dumping, or changes in taste or smell. These questions were asked as a structured yes/no item with a free-text "please specify" field. Additional supplement-associated complaints reported in the free-text field were subsequently coded into categories by the research team before analysis.

During the first contact, all patients were advised not to take the supplement on an empty stomach and to take the supplement with a meal, preferably lunch or dinner. To promote slower dissolution, patients were advised to use the chewable tablet instead of the capsule, to split the chewable tablet into two halves taken with lunch and dinner, and to suck the tablet rather than chew it. They were also advised to take the chewable multivitamin separately from the iron/copper tablet.

The complete call script is presented in Appendix B. The structured intake advice evaluated in this study consisted of five components that were delivered to all 116 initially contacted participants as part of the standard package at the first contact (as mentioned above). The call script also contained additional escalation recommendations, namely opening the capsule and mixing the contents with compote, the use of warm beverages or ginger tea, and a temporary pause in supplementation, which were intended only for the subset of patients in whom nausea persisted despite the standard advice. Because these escalation recommendations were not applied uniformly across the cohort and the subset of patients with persistent nausea at follow-up was small (n=20), patient-reported helpfulness of the escalation options is outside the scope of the present analysis. The version of the call script presented in Appendix B reflects the items that were consistently completed across the cohort, and that formed the analytic dataset; it has been edited from the broader FitForMe customer-service template used during routine support interactions.

During the second contact, a follow-up call was scheduled one week after initial contact, to assess whether nausea persisted and to evaluate the patient-reported effect of the advice given. This was done using structured questions on nausea status, effectiveness of advice, and perceived changes in symptoms. Specifically, nausea resolution was operationalized as a structured binary question (“Are you still experiencing nausea?”: “yes/no”) asked during the follow-up telephone call. The perceived helpfulness of each individual recommendation was subsequently assessed using separate yes/no questions for each component of the bundled advice.

This investigation was conducted as a service evaluation within FitForMe's routine customer-support activities and was not designed as a prospective research study; participants received supportive guidance and follow-up contact that were part of standard customer care, and participation was voluntary. Because the activity did not impose any experimental procedure on participants, it fell outside the scope of the Dutch Medical Research Involving Human Subjects Act (WMO), and review by an accredited Medical Ethics Review Committee was therefore not required. Written research consent was consequently not obtained at the time of data collection. Verbal consent for participation in the supportive guidance and follow-up contact was obtained from each customer at the start of the initial telephone contact, and reconfirmed before the follow-up call one week later. The dataset analyzed for this publication was fully anonymized before any analysis, in line with independent legal counsel. Under EU General Data Protection Regulation Article 4 and Recital 26, the processing of anonymized data falls outside the material scope of the regulation.

Analyses

All statistical analyses were conducted using IBM SPSS Statistics version 29.0.2.0 (released 2022; IBM Corp., Armonk, New York, United States). Categorical variables are presented as frequencies with percentages. For paired categorical variables, changes over time within the same patients were assessed using the McNemar test. Statistical significance was defined as p<0.05.

## Results

Part one: survey

**Figure 1:**
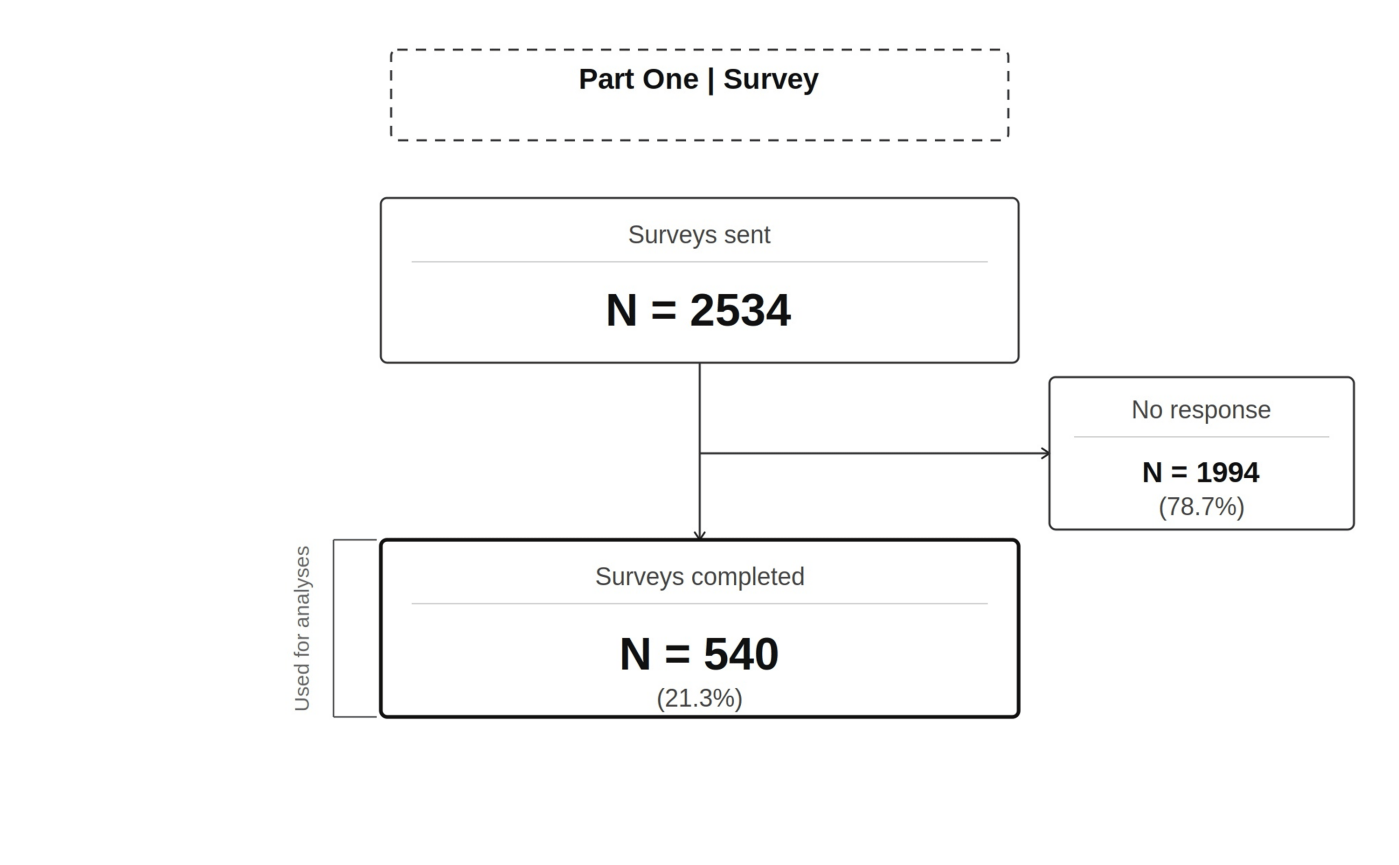
Participant flow for part one. Of the 2,534 customers contacted by email, 540 (21.3%) completed the online survey regarding nausea associated with bariatric multivitamin use.

An overview of participant flow is presented in Figure 1.

The study population consisted of 540 patients from eight commercial regions covering 10 countries, predominantly France (n=204, 37.8%) and the Netherlands (n=155, 28.7%). Most patients underwent sleeve gastrectomy and used the Optimum supplement (n=346, 64.1%), whereas the remaining patients underwent Roux-en-Y gastric bypass and used the Forte supplement (n=194, 35.9%). In total, 431 patients (79.8%) took the supplement in capsule form, whereas 109 patients (20.2%) used the chewable version.

A comprehensive overview of nausea onset, triggering factors, moment of occurrence, and impact on daily functioning is provided in Table 1.

**Table 1:** Descriptive statistics for the timing and impact of nausea (n=540)

Domain	Response option	n (%)
Start of supplement intake	Start before surgery	94 (17.5)
	Start right after surgery	85 (15.7)
	Start 2-7 days after surgery	103 (19.1)
	Start 8-14 days after surgery	133 (24.6)
	Start at another moment	125 (23.1)
Timing of supplement intake during the day	Before breakfast	57 (10.6)
	During a meal	128 (23.7)
	Between two meals	124 (23.0)
	Before going to bed	75 (13.9)
	Other intake moment	156 (28.8)
Onset of nausea	As soon as the supplement started	356 (65.9)
	Within the first few months	66 (12.2)
	After a few months	32 (5.9)
	After more than a year	23 (4.3)
	Unknown onset	63 (11.7)
Nausea triggers	Seeing the supplement	25 (4.6)
	Smelling the supplement	68 (12.6)
	Tasting the supplement	65 (12.0)
	After swallowing	275 (50.9)
	Slow dissolving	18 (3.3)
	Other trigger	89 (16.5)
Timing of nausea around intake	Before intake	27 (5.0)
	Right after intake	122 (22.6)
	Two minutes after intake	75 (13.9)
	Five minutes after intake	80 (14.8)
	10 minutes after intake	128 (23.7)
	30 minutes after intake	67 (12.4)
	One hour after intake	10 (1.9)
	>One hour after intake	3 (0.6)
	No answer given	28 (5.2)
Restriction of daily life (0-10 scale)	Score 0 (no restriction)	35 (6.5)
	Score 1	20 (3.7)
	Score 2	70 (13.0)
	Score 3	18 (3.3)
	Score 4	32 (5.9)
	Score 5	35 (6.5)
	Score 6	57 (10.6)
	Score 7	46 (8.5)
	Score 8	91 (16.9)
	Score 9	91 (16.9)
	Score 10 (unable to do anything)	45 (8.3)
Impact description	Symptoms reduce over time	210 (38.8)
	Uncomfortable but not limiting	97 (18.0)
	Interferes considerably	145 (26.9)
	Unable to perform daily activities	88 (16.3)

Part one demonstrated that incorrect or potentially suboptimal intake practices were common in this selected cohort, with 256 patients (47.4%) taking the supplement on an empty stomach (i.e., before breakfast, between meals, or before bedtime) and 188 patients (34.8%) initiating supplementation within seven days after surgery, a period during which solid food is generally not yet consumed. The majority (n=356, 65.9%) experienced nausea as soon as they started taking the supplement. Nausea was triggered even by seeing or smelling the supplement in 93 patients (17.2%). Complaints were experienced within 30 minutes after supplement intake by 472 (87.4%) patients. Overall, 365 patients (67.6%) reported a score of 5 or higher on a 0-10 scale measuring the impact on daily life, where 0 indicated no restriction, and 10 indicated being unable to do anything.

Part two: follow-up after intake advice

**Figure 2:**
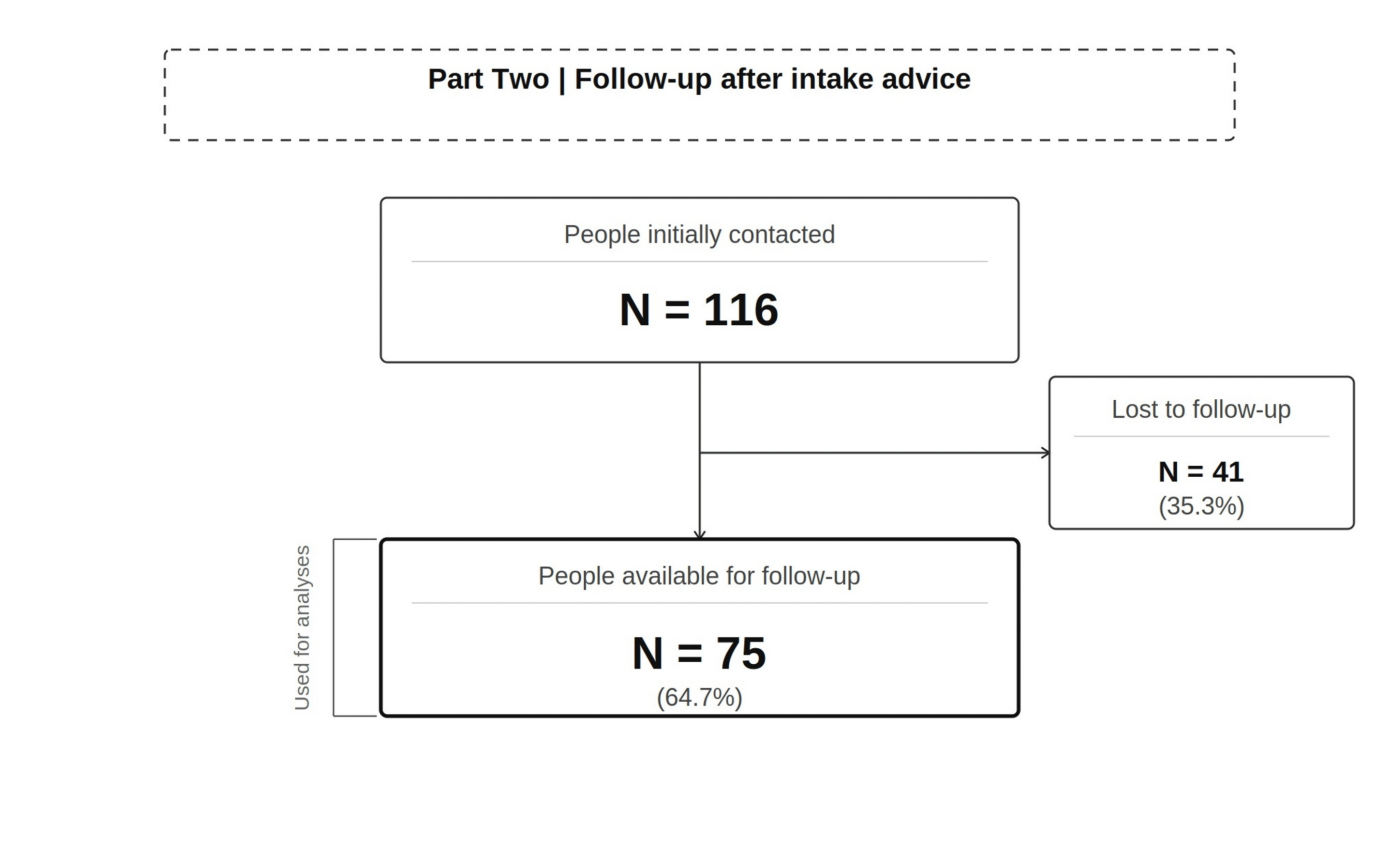
Participant flow for part two. Of the 116 customers contacted by telephone, 75 (64.7%) were available for follow-up one week after receiving structured intake advice and were included in the analysis.

An overview of participant flow is presented in Figure 2.

In a separate sample of 116 patients contacted, 75 patients (64.7%) were available for the follow-up call. All 116 patients received structured intake instructions, and 75 patients (64.7%) were available for follow-up and were included in the analysis. Of the 116 patients initially contacted, 41 (35.3%) could not be reached for the scheduled follow-up call and were therefore excluded from the follow-up analysis. As no further data could be collected for this subgroup, a comparison with the 75 patients included in the analysis was not feasible.

Of the 75 patients, 44 (58.7%) used a capsule and were sent chewable tablets, and at follow-up, all patients (100%) tried the chewable tablets.

At baseline, all patients reported nausea, and 62 patients (82.7%) reported experiencing one or more other supplement-associated complaints in addition to nausea, including stomach pain, diarrhea, bloating, vagal discomfort, sweating, hot flashes, chills, hypersalivation, dizziness, tingling, headache, and fatigue. Dumping symptoms related to food intake were reported by 30 patients (40.0%), food intolerances by 28 (37.3%), and changes in taste following surgery by 31 (41.3%).

In the follow-up cohort, 55 patients (73.3%) reported resolution of nausea one week after receiving structured intake advice.

Changing from capsule to chewable tablet was reported as a significant (p<0.001) improvement in 41 patients of the 44 patients who switched from capsule to chewable (93.2%; 54.7% of the total follow-up cohort, n=75; p<0.001). Other adjustments were also reported as having a significant (p<0.001) positive effect: taking the supplement with lunch and dinner (n=42, 56.0%), sucking the tablet (n=20, 26.7%), or cutting the tablet in half (n=20, 26.7%). Taking the iron/copper tablet separately from the multivitamin was reported as effective in one patient (p=1.000) (Table 2).

**Table 2:** Effect of supplement intake adjustments on nausea All participants (n=75) reported nausea before receiving advice. As part of the advice, all 75 participants switched to, or continued using a chewable tablet. The "switch from capsule to chewable" recommendation was directly applicable to the 44 patients (58.7%) using a capsule at baseline; the remaining 31 patients (41.3%) were already using the chewable formulation. "Good effect" reflects the proportion of patients who endorsed the individual component as having reduced their nausea at the one-week follow-up.

Advice	Applicable n	Good effect n (%)	p-value
Not on an empty stomach (taken with lunch/dinner)	75	42 (56.0)	<0.001
Switch from capsule to chewable	44	41 (93.2)	<0.001
Suck the tablet	75	20 (26.7)	<0.001
Cut the tablet in half	75	20 (26.7)	<0.001
Take separately from iron	75	1 (1.3)	1.000

## Discussion

This study provides insight into patient-reported nausea associated with bariatric multivitamin use and into symptom changes reported after structured intake advice in a selected follow-up cohort. These findings suggest that several practical intake-related factors may influence tolerability, including taking supplements with a meal, using chewable formulations, and slower dissolution. At the same time, symptom patterns reported by patients suggest that nausea after MBS is often multifactorial and not solely attributable to the supplement itself.

Possible causes of nausea

Dumping May Be Mistaken for Supplement-Related Nausea

More than 80% of the patients in this study reported supplement-related complaints similar to dumping symptoms, and 40% reported experiencing classic food-related dumping. This overlap is noteworthy; the molecules in highly dosed multivitamins may theoretically contribute to early dumping through osmotic shifts. Further research is necessary to evaluate this potential mechanism. The timing of symptoms aligned with early dumping, given that 275 patients (50.9%) experienced complaints between 5 and 30 minutes after supplement intake. However, because supplements are often taken with or right after a meal, many patients may mistakenly attribute their nausea to MVS, when in fact they could be experiencing symptoms of classic dumping syndrome triggered by their meal.

Fear Conditioning and Avoidance Behavior

Fear conditioning and avoidance behavior are significant challenges in the management of post-bariatric supplementation. For example, an initial episode of nausea shortly after surgery or as part of early dumping symptoms may coincide with multivitamin use, leading patients to form a negative association between the supplement and their symptoms. Over time, this can lead to a conditioned response, where the supplement itself, or even the thought of taking it, triggers nausea independent of any physiological cause. In the present study, 29.3% of patients reported getting nauseous by seeing, smelling, or tasting the supplement. This finding indicates that, for these patients, nausea is unlikely to be triggered by physiological mechanisms and appears more consistent with psychological, conditioned, or associative responses. This phenomenon is well-described in the context of chemotherapy-induced nausea [8]. Moreover, a study by Nguyen et al. found that pregnant women often avoided taking multivitamins due to previous experiences or fears of nausea and vomiting [9]. This may also apply to bariatric patients who experience early postoperative gastrointestinal discomfort. After one or more negative experiences (e.g., nausea), patients may develop avoidance behavior. This interpretation remains speculative and should be tested in future prospective studies.

Iron and Nausea

Although iron supplementation is commonly associated with gastrointestinal side effects, such as nausea in athletes [10], pregnant women [11], and menstruating women [12], the strength of this association varies, and evidence quality is often low or inconsistent, especially in non-anemic individuals. In the present dataset, there was no clear descriptive pattern suggesting that iron content alone explained the reported nausea, although this observation should be interpreted as descriptive rather than as evidence against an iron-mediated effect. Only one patient specifically reported relief upon separating the iron/copper tablet from the chewable multivitamin, which is too few to support any conclusion in either direction. The contribution of iron to supplement-related nausea in this cohort, therefore, remains uncertain and warrants targeted prospective evaluation.

Clinical implication

These findings suggest that small, simple changes to supplement intake, such as adjusting the timing or formulation, may be associated with improved tolerability in bariatric patients.

The overlap between supplement-related nausea and dumping symptoms highlights the importance of distinguishing between the two phenomena. Clinicians should be aware of this potential confusion and ensure that patients are properly educated about the differences between supplement-related symptoms and early dumping syndrome. By understanding these distinct causes, healthcare providers can better address nausea complaints and optimize patient care.

These findings may support clearer counseling on multivitamin intake practices in both clinical and commercial support settings. Prospective studies are needed to determine whether standardized intake counseling improves long-term adherence or nutritional outcomes.

Limitations 

This study has several limitations.

First, the observed 73.3% nausea-resolution rate cannot be causally attributed to the structured intake advice. The study had no control group, and a no-treatment or alternative-treatment control was not feasible in this real-world setting. Participants contacted customer service specifically because of nausea and were seeking immediate help; so, withholding advice was neither ethically nor practically appropriate, while providing different advice would still constitute an active intervention rather than a true control condition. As a consequence, natural resolution of symptoms cannot be excluded as a contributing explanation, because early postoperative nausea is known to improve spontaneously in a substantial proportion of bariatric patients. In addition, the advice consisted of multiple simultaneous recommendations, which limited our ability to isolate the independent effect of each individual component, although patients were subsequently asked which advice they perceived as helpful. The structured advice to take supplements with meals may have further prompted concurrent changes in meal timing, regularity, or food intake, factors that can independently influence dumping-related symptoms (40.0%) and food intolerance (37.3%). Finally, data collection and follow-up calls were performed by FitForMe-employed dietitians, and complimentary chewable samples were provided at the first contact. While this approach mirrors the real-world customer-care pathway under evaluation and supports external validity, it may also have introduced social desirability bias and expectancy effects. Taken together, the 73.3% nausea-resolution rate reflects patient-reported outcomes within a commercial customer-service context rather than effect estimates obtained under independent or blinded conditions, and the contribution of spontaneous recovery, individual advice components, and modified eating behavior cannot be quantified within the present uncontrolled design. From a practical standpoint, however, this does not diminish the value of the advice, as patients reported clinically meaningful resolution regardless of the exact mechanism through which it arose. The purpose of this real-world study was to evaluate the effect of the complete package of standard advice used in routine customer care.

Second, selection and response bias may affect generalizability. The study population was selected, as it included only customers who contacted FitForMe because of nausea; the findings are therefore not generalizable to the broader bariatric population or to users of other supplement formulations. However, representing the entire bariatric population was not the aim of this study; it specifically targeted the subgroup of patients who experienced nausea while using a specialized bariatric multivitamin, as this was the population of primary practical relevance. In addition, response rates were 21.3% in part one (540 of 2,534 invited) and 64.7% in part two (75 of 116 contacted), which are realistic engagement levels for voluntary, telephone-based service-evaluation settings but introduce potential selection and attrition bias. Non-responders may have differed systematically, for example, by experiencing more severe symptoms or having already discontinued supplementation, and the 73.3% nausea-resolution rate therefore applies specifically to the 75 follow-up completers rather than to all initially contacted patients.

Third, outcomes were assessed using subjective, non-validated measures rather than standardized symptom scales. This may have introduced measurement bias and limited reproducibility. However, these outcomes were collected in the context of routine customer service interactions, reflecting the pragmatic real-world design of the study rather than a formal clinical research setting.

Fourth, follow-up duration was limited to one week after providing intake advice, and the long-term impact on nausea was not assessed. Nevertheless, the short-term findings are clinically relevant, as most participants (65.9%) reported that nausea started when they began taking the supplements, suggesting a direct relationship with supplement intake. If inadequate intake behavior contributed to these symptoms, an early resolution after providing proper intake instructions would be plausible. The observed short-term effect therefore supports a promising early benefit; however, longer follow-up is needed to determine whether this is sustained and improves long-term tolerability and adherence.

Fifth, important clinical variables, such as medication use, postoperative complications, and nutritional or laboratory status, were not available in the dataset. Because this was a real-world observational study based on routine customer service contacts, these variables were not systematically collected, as they are not part of standard customer questioning.

Strengths

This study has several strengths. First, this study addresses a gap in the adherence literature, which has predominantly focused on forgetfulness, cost, and access as drivers of non-adherence. By characterizing tolerability as a distinct and clinically relevant driver of non-adherence after bariatric surgery and by identifying simple, modifiable behavioral adjustments that can mitigate it, this work broadens the conceptual and practical scope of how supplement adherence in this population may be supported. Second, it reflects a real-world population of bariatric patients who actively sought support because of nausea, enhancing the practical relevance of the findings for routine care and customer support settings. Third, the study addressed a clinically meaningful subgroup of patients at risk of discontinuing a specialized bariatric multivitamin because of tolerability concerns. Fourth, intake advice was delivered in a structured and consistent manner by trained dietitians, allowing a standardized evaluation of patient-reported symptom changes after counseling. Finally, although the follow-up period was limited, the one-week assessment was appropriate for evaluating the immediate impact of routine intake advice on early tolerability, which is highly relevant when symptoms arise soon after supplement initiation.

## Conclusions

Customers contacting FitForMe with nausea complaints received simple, structured intake instructions designed to ensure a more gradual release of vitamins and minerals from WLS Forte or WLS Optimum on a full stomach. In the follow-up cohort, many participants subsequently reported resolution of nausea. Switching from a capsule to a chewable tablet and changing the intake behavior were commonly reported as helpful strategies. However, this study also suggests that supplement-related nausea is often multifactorial and not solely driven by the supplement itself. By recognizing modifiable factors, healthcare providers and supplement manufacturers may be able to support tolerability and long-term adherence to supplementation, a critical component of post-bariatric care.

These conclusions apply specifically to bariatric patients who experience nausea during use of FitForMe specialized multivitamins, WLS Optimum, and WLS Forte, and proactively seek support. They should not be extrapolated to the general post-bariatric population, to asymptomatic patients, or to other multivitamin formulations without further evaluation. While the reported resolution rates should be interpreted with caution, given the uncontrolled real-world design, and confirmation in prospective controlled studies remains warranted, the recommendations themselves are simple, low-cost, and low-risk. Adjustments to intake timing, formulation, and dosing can be readily incorporated into routine post-bariatric counseling by healthcare providers and easily applied by patients at home, and may therefore be considered a reasonable pragmatic first step in clinical practice while more definitive evidence is awaited.

## Appendices

Appendix A: nausea survey

Country, surgery type, and supplement formulation were retrieved from FitForMe's customer records via the unique customer ID. The survey was translated into the native language of each country.

1. When did you start taking the multivitamin?

☐ Before the surgery

☐ Immediately after the surgery

☐ Two to seven days after the surgery

☐ 8-14 days after the surgery

☐ Other, __________________________

2. When do you take your FitForMe multivitamin?

☐ Before breakfast

☐ During a meal

☐ Between two meals

☐ Before going to bed

☐ Other, __________________________

3. When did the nausea complaints start?

☐ As soon as I started taking the supplements

☐ In the first few months of using the supplements

☐ After a few months of use

☐ After more than a year of use

☐ I don’t know exactly, but approximately ____ months after the surgery

4. Which statement best applies to you?

☐ I already feel nauseous when I see the multivitamin

☐ I feel nauseous when I smell the multivitamin

☐ I feel nauseous when I taste the multivitamin

☐ I only feel nauseous after swallowing the multivitamin

☐ I feel nauseous because the multivitamin goes down slowly

☐ Other, __________________________

5. At what moment do you start feeling nauseous from the multivitamin?

☐ Before intake

☐ Immediately after intake

☐ Two minutes after intake

☐ Five minutes after intake

☐ 10 minutes after intake

☐ 30 minutes after intake

☐ One hour after intake

☐ More than one hour after intake

6. Describe your feeling of nausea in a few words (e.g., sick feeling, gagging, sweating): ______________________________

7. On a scale of 0 to 10: to what extent does the nausea limit you in daily life? 0 = no restriction | 10 = unable to do anything _____ / 10

8. Which statement best describes your nausea complaints?

☐ After taking the capsule/chewable tablet, you feel nauseous, sometimes with gagging or vomiting. The complaints subside after some time, and you can continue daily activities.

☐ Nausea is nagging and unpleasant, but does not interfere with most daily activities. Distraction helps somewhat.

☐ You experience gagging, vomiting tendency, and/or stomach pain. Nausea interferes significantly with daily activities and requires lifestyle adjustments.

☐ You regularly vomit and have stomach pain. Nausea prevents you from performing daily activities, and you cannot function normally.

9. What have you tried to reduce nausea complaints? (check all that apply) ☐ Swallow with water

☐ Swallow with juice

☐ Swallow with yoghurt

☐ Swallow with coffee

☐ Pinch nose while swallowing

☐ Open capsule/grind the chew tablet and mix with food

☐ Intake at different moments of the day

☐ Switch from capsule to chew or vice versa

☐ Other: __________________________

10. Which of the options you tried worked best for you? ☐ Swallow with water

☐ Swallow with juice

☐ Swallow with yoghurt

☐ Swallow with coffee

☐ Pinch nose while swallowing

☐ Open capsule/grind the chew tablet and mix with food

☐ Intake at different moments of the day

☐ Switch from capsule to chew or vice versa

☐ Other: __________________________

11. What do you think is the best advice FitForMe can give to help reduce these nausea complaints? ______________________________

12. Do you have any other comments, questions, or suggestions? ______________________________

Appendix B: nausea call script

Note on translation: The original call script was developed and used in French during routine FitForMe customer service interactions in France. The version presented here has been translated into English by the authors for the purpose of this publication. The translation aims to faithfully reflect the structure, content, and clinical intent of the original French script; minor linguistic adaptations were made to ensure readability in English.

First Contact

Customer Information

Customer ID: __________________________

Date of contact (phone call): __________________________

Date of surgery: __________________________

Type of surgery:

☐ SG

Date started with FitForMe: __________________________

Intake Form

☐ Chew

☐ Capsule

When did the nausea start after beginning FitForMe?

☐ Immediately with the first intake

☐ After one to three days

☐ After three to six days

☐ After one week

☐ After one month

☐ Longer than one month

Please describe symptoms; __________________________

Over the past two weeks, have you experienced nausea when taking certain kinds of food without taking the supplement?

☐ No

☐ Yes (please specify): __________________________

Over the past two weeks, in case you experienced nausea, did your heart rate go up; did you have abdominal pain or cramps; did you feel dizzy, etc.?

☐ No

☐ Yes (please specify): __________________________

After your surgery, did your taste or smell change?

☐ No

☐ Yes, how did it change? __________________________
 

General Advice for Customers With Nausea* (provided to all customers initially contacted (n=116))

* Do not take it on an empty stomach (i.e., not before breakfast, not between meals, and not before going to bed). Take the supplement together with a meal, preferably lunch or dinner.

* If applicable, switch to a chewable tablet.

* Break the chewable tablet in half: take one half with lunch and the other half with dinner.

* Let the tablet dissolve slowly in the mouth rather than chewing and swallowing it quickly.

* Take the chewable multivitamin separately from the iron/copper tablet (i.e., at a different moment of the day, or with a different meal).

Follow-Up

Date of follow-up contact: __________________________

Are you still experiencing nausea?

☐ No

☐ Yes

Which advice helps reduce nausea?

☐ Not on an empty stomach

☐ Suck on the tablet

☐ Break chew the tablet in half

☐ If applicable, switch intake form (e.g., to chewable)

☐ Take without iron

☐ Open capsule

☐ Other: __________________________

Only for people who took the chew tablet: Did you experience nausea when taking the chew tablet, or did you experience nausea when taking the separate iron tablet?

☐ Nausea after the chew tablet

☐ Nausea after the separate iron tablet

☐ Nausea after both
